# Models of global primary care post-2030

**DOI:** 10.1016/j.lanprc.2025.100027

**Published:** 2025-09

**Authors:** Luke N Allen, Kumanan Rasanathan, Robert Mash, Manuela Villar Uribe, Viviana Martinez-Bianchi, Michael Kidd

**Affiliations:** a Nuffield Department of Primary Care Health Sciences, University of Oxford, Oxford, UK; b Alliance for Health Policy and Systems Research, World Health Organization, Geneva, Switzerland; c Department of Family and Emergency Medicine, Stellenbosch University, Cape Town, South Africa; d Health, Nutrition and Population Global Practice, The World Bank, Washington, DC, USA; e Department of Family Medicine and Community Health, Duke University School of Medicine, Durham, NC, USA; f International Centre for Future Health Systems, University of New South Wales, Sydney, NSW, Australia

## Abstract

Primary care is currently a central focus in global health policy; however, renewed attention has not translated into the investment needed to build systems that are fit for the future. As 2030 approaches, many health systems are converging towards primary care models that provide community-based, first-contact access, but they omit the other core functions of comprehensiveness, continuity, and coordination. In this Viewpoint, we argue that these primary care lite models are ill-equipped to manage the increasing burden of multimorbidity; harness technological disruption; and reduce health inequities. We propose a new trajectory towards hybrid models of care that anchor community-oriented outreach workers within multidisciplinary teams that are trained in family medicine. Although artificial intelligence and digital tools can magnify impact and reach, we warn that their uninformed adoption could create digital gatekeepers and deepen disparities. To future-proof primary care, policy makers should invest in integrated models that deliver robust, equitable, and person-centred care that is needed to meet future challenges.

## The countdown to 2030

To meet the complex health challenges of the coming decades, the global health community should reject minimalist approaches to primary care and invest in models capable of delivering the core functions of first-contact access, comprehensiveness, coordination, continuity, and person centredness,^1^ built on the foundations of family medicine. Deviation from this standard risks establishing a two-tier system of strong primary care for the privileged and primary care lite for those living in poverty.

In 2015, world leaders agreed on a sustainable development agenda for the next 15 years, comprising 17 Sustainable Development Goals (SDGs) accompanied by 169 unique targets.^2^ We are currently in the final years of the countdown to 2030, and although there has been remarkable progress in some areas of SDGs, the world is not on track to meet many of the health-related SDGs.^3^ The WHO Director-General has repeatedly stated that universal health coverage (UHC) is the centrepiece of the SDG health targets.^4^ However, the expansion of service coverage has stalled, health-related financial strain has increased, and over 4·5 billion people still lack access to many essential health services.^3^

The looming 2030 deadline has ignited global interest in strengthening primary care and the primary health care (PHC) approach (panel 1) as the fastest and most effective, equitable, and inclusive path to UHC.^3^ Hospital-based services are indispensable; nevertheless, the vast majority of people access essential services through primary care.^8^ WHO estimates that primary care can deliver over 90% of essential health services and generate three-quarters of the projected SDG health gains.^9^ Primary care providers in countries such as Costa Rica, Brazil, Thailand, and South Africa have shown that well-supported, family medicine-led models can improve access and equity to primary care services and health outcomes.^5^^,^^10^

Panel 1 **Primary care is a subset of primary health care (PHC)5–7**

Normatively, primary care is a clinical service delivery platform that manages people presenting with undifferentiated symptoms and provides promotive, protective, preventive, curative, rehabilitative, and palliative care throughout the life course. Primary care providers are based in local communities, close to where people live and work. There are five core functions of primary care, commonly referred to as the 5 Cs:

• First-contact accessibility: ensures timely, equitable access
• Continuity: builds trusted relationships and coherent clinical management over time
• Coordination: supports service integration, guiding people through different providers and levels of care to receive appropriate services
• Comprehensiveness: provides a full range of services, matched to local needs
• Person centredness: works in partnership with people to tailor care to their unique needs and preferences and empowers people to take an active role in their health

Not all primary care realises this comprehensive vision, but evidence and experience show that the vision is possible. Although strong primary care can be delivered through various configurations of health workers, there is growing evidence that teams supported by clinicians with postgraduate family medicine training are more likely to deliver on the five core functions. Family medicine specialists are trained to provide comprehensive, continuous, and coordinated care across the life course, often with a strong emphasis on prevention, community engagement, and management of multimorbidity. These skilled generalists balance individualised clinical decision making with population health goals and work effectively in multidisciplinary teams—often supervising networks of first-level providers. Although not every setting might have the resources to deploy family physicians at scale, their inclusion can greatly enhance the quality, equity, and scope of primary care services.

Primary care is a subset of the much broader concept of PHC, a philosophical all-of-society approach to organising and strengthening health systems. PHC emphasises three components: multisectoral policy and action to address social and environmental determinants; community empowerment; and integrated health services based on strong primary care and essential public health functions. The seminal Declaration of Alma-Ata outlines a series of PHC values, including solidarity, equity, mutual responsibility, social justice, and the human right to health.

Primary care now features in the strategy documents of the Global Fund to Fight AIDS, Tuberculosis and Malaria, Global Financing Facility, World Bank, and various other health and development partners.^11^ The *Lancet Global Health* Commission on financing primary health care called for an additional US$200–328 billion per year to strengthen primary care in low-income and middle-income countries (LMICs),^12^ and the WHO 2016–30 Global Strategy on Human Resources for Health called for a paradigm shift, reorienting health systems towards a collaborative primary care approach.^13^

Arguably, primary care has not received this level of attention since the 1978 Declaration of Alma-Ata.^14^^,^^15^ Yet the renewed policy focus has not translated into meaningful shifts in resources. According to WHO health financing data, primary care might deliver 90% of all services, yet it receives less than 10% of total health expenditure.^16^ Furthermore, compared with hospital-based specialities, primary care is substantially under-represented in terms of research funding and academic professorships. Another pressing concern is that health systems in both high-resource and low-resource settings are converging towards a limited model of primary care that relies heavily on early-career conscript doctors (as seen in parts of South America), non-specialist general practitioners, and mid-level staff who lack adequate supervision, infrastructure, and resources needed to deliver coordinated, continuous, comprehensive, and person-centred care.

It is clear that in the future, our current models of primary care delivery will face major disruptions in the face of demographic, epidemiological, environmental, social, and political change. Factors such as artificial intelligence (AI), climate change, ageing, and political polarisation are transforming our societies. Technological disruption is creating new opportunities to extend access to services while also raising fresh challenges around safety, equity, and accountability. Although the salience of a post-2030 global agenda might have diminished in the context of the current geopolitical zeitgeist, our increasingly multipolar world characterised by regional alliances will still need strong, efficient, and equitable primary care to meet the challenges of the future. Primary care will need to reshape to adapt to these trends and deliver on its potential, while remaining true to the principles of Alma-Ata.^14^

## Future challenges

In the words of Neils Bohr, “prediction is very difficult, especially if it is about the future”.^17^ Yet many of the major challenges, disruptions, and innovations that will shape the next 20 years are already in motion today. Global health threats from ecological crises, political ruptures, emerging infectious disease outbreaks, and a worrying uptick in conflict and violence might continue their recent trend of increasing in frequency.^18^^,^^19^ The global burden of disease will increasingly be dominated by non-communicable diseases and mental health conditions, which are already the leading causes of service demand and disability in many countries.^20^ The global demographic transition will continue, doubling the number of people older than 60 years.^21^ Rising multimorbidity, polypharmacy, new medical technologies, and increasing old-age dependency ratios will combine to put enormous financial strain on health systems.^22^ Other persistent macrotrends include underfunding by governments, private sector, and philanthropic agencies on essential primary care services; geographical barriers in rural areas; poor care quality; the rise of antimicrobial resistance; and the spread of misinformation and disinformation.

As seen over the past 20 years, the key challenge of health inequity potentially lies within countries, with gaps between groups widening. In contrast, the average gap between LMICs and high-income countries (HICs) has reduced through rapid gains in life expectancy. However, this trend is not guaranteed to continue. Conflicts, environmental catastrophes, and acute collapse of official development assistance might disproportionately affect low-income countries. Within the health sector, widening inequities could also be driven by differential health gains from personalised, genomic, and AI-augmented medicine disproportionately benefiting individuals with the ability to pay, while poorer and marginalised populations continue to face systemic barriers to access.^23^, ^24^, ^25^ The current unequal access to transformative therapies for cancer, cardiovascular disease, and diabetes—largely limited to wealthy individuals in wealthy countries—is ominous in this regard.

Digitalisation and AI further complicate the picture. Digital health records governed and carried by people themselves promise to empower people and provide a decentralised solution to the fragmentation of health systems.^26^ In the near future, the highest quality primary care will be offered by human clinicians augmented with AI support for diagnosis, coordination, and evidence-based therapy.^27^ General-purpose AI-only service delivery models may not be as good as complementary human–digital models of care, especially in the near-term, but they might represent a considerable improvement for very low-resource and rural settings in which existing levels of provision are absent or very weak.^28^ However, there is a potential risk that some disadvantaged communities might be offered AI-only care. In such scenarios, a digital gatekeeper might replace the first point of contact with a human clinician. Furthermore, the implicit biases in large language models used for AI-based care could further undermine the experience and quality of care for marginalised groups and establish structural discrimination within health systems.^29^ Guardrails, accountability, and equity-based governance will be crucial to ensure that AI addresses rather than increases inequalities.

Positive trends include the growing emphasis on equitable access to care; the enormous positive potential of AI for primary care; the continued rise of genomics and personalised medicine; growing interest in people-centred care; and WHO-led efforts to reorient health systems around the principles of PHC. New technologies and ways of working help individuals to take control of their primary care within their home setting, by deploying resources and information for their empowerment and improved outcomes. Beyond health, UN member states are advancing commitments on climate change, inequality, gender equity, governance, and digital transitions through platforms such as the 2024 Pact for the Future.^30^ Although this document calls for “turbocharging progress towards achieving the SDGs by 2030 and beyond”, the global appetite to pursue another major development agenda after the SDGs remains uncertain, given that global solidarity is currently weakening. However, the reaffirmed commitment to UHC in the Pact for the Future provides an opportunity to reconsider the role of primary care in addressing the challenges of the coming decades.

Primary care can be the engine powering advances towards UHC and is central to tackling emerging health threats. Primary care also contributes to managing increasing multimorbidity, detecting and responding to new outbreaks, stewarding antimicrobials, responding to climate-driven health challenges, building trusting community relationships, and deploying AI-augmented care. As new providers and technologies enter the health-care ecosystem, primary care’s coordination role will become ever-more valuable in advocating for people, helping them to navigate services, integrating their care, and collating and interpreting their data and results from across a wide range of platforms and providers. As the first point of entry into the health system for all non-emergency care and the provider of virtually all essential health services, health system strengthening initiatives are rightly focused on bolstering primary care capacity and resilience. To effectively operate as the health system’s first line of defence, we need strong supportive structures (eg, governance and financing) and inputs (ie, workforce, medicines and supplies, and infrastructure). However, we also need to consider whether the current dominant models of care are fit for the future. This consideration is especially true as recent high-profile PHC frameworks and monitoring tools have tended to stress financing arrangements, policies, medicines, supplies, facilities, and services, arguably at the expense of models that deliver the core components of primary care.^31^^,^^32^

## Dominant contemporary models of care

In the years following the Declaration of Alma-Ata, a group of western development funders and health agencies struggled with the tractability of PHC and elected to switch to a limited set of objectives at the seminal 1979 Bellagio conference convened by the Rockefeller Foundation.^33^ The admittedly ambitious whole-of-society vision was reduced to so-called selective primary care, comprising community-based delivery of only four interventions: growth monitoring, oral rehydration, breastfeeding, and immunisations.^33^ We risk repeating the same reductive mistakes with primary care services, narrowly focusing on the delivery of a modest basket of essential services while de-prioritising the strengthening of the five core functions that are essential to meeting challenges ahead.

Despite the confusing array of primary care models—varying across urban and rural settings, income levels, and degrees of public, private, and hybrid provision—three broad group models can be identified based on who provides the majority of first-contact care in the community. The first model includes family physicians (called general practitioners in countries such as the UK and Australia) predominantly present in HICs where they work from health centres that serve most communities, often supported by multidisciplinary teams. The second model includes non-specialist doctors and mid-level providers such as general practitioners in many LMIC settings (who, unlike in the UK and Australia, do not have postgraduate specialist training in family medicine, eg, across most of India and Africa) or nurse practitioners, physician assistants or clinical officers, and pharmacists. Community health workers (CHWs) often provide additional first-contact care for selected conditions, extending the reach of health centres. The third model includes organ-specific specialists such as cardiologists and gynaecologists who often serve as the first point of contact (rather than a specialist generalist) in former Soviet states and some Middle Eastern countries.

The three broad models have emerged from differing historical, political, and economic trajectories. Primary care models are ultimately shaped by how services are funded and governed. In many countries, the private sector contributes substantially to primary care delivery—especially in urban LMIC settings. Donor funding, social health insurance, and out-of-pocket payments all influence care access, continuity, and coordination.

Family physician-led systems often evolved alongside welfare-state expansions in western Europe and settler–colonial states, embedding general practice within UHC frameworks. The increase in the number of mid-level providers reflects both task-shifting imperatives and workforce shortages, particularly in rapidly urbanising middle-income contexts. Meanwhile, the organ specialist model in former Soviet states stems from the Semashko tradition, which favoured disease-specific verticality and extensive hospital networks over generalist, community-based care. Different overlapping models co-exist in many countries; for instance, Uganda has specialist family physicians operating alongside many other different providers of first-contact care. The ability of each of these cadres to deliver the core functions of primary care varies widely, especially in the absence of multidisciplinary teams.

All three broad models offer first contact in the local community and can offer relational continuity. Informational and management continuity are dependent on team composition and the use of medical records. Coordination involves referrals, and importantly, counter-referrals—such as feedback integration from multiple other specialists and services to continually optimise each patient’s care and maintenance of a holistic overview of their different health needs and progress through other parts of the health system. Person centredness involves tailoring management to meet the needs of individual people, working in partnership with them, and providing bio-psycho-social care. We feel that models that use health workers trained in family medicine and include multidisciplinary teams are the most likely to be delivering the core functions of primary care and are best positioned to face the challenges of the future compared with models based on independent practitioners with limited family medicine training. For example, in many settings, nurse practitioners are the main primary care providers, but they are rarely trained or resourced to develop a family medicine mindset in which they take responsibility for the holistic care of people over time.

## Delivering the core functions of primary care in every community

Although many countries are increasingly using CHWs to extend access to primary care, there is a risk that too much responsibility is being placed on this cadre. There is increasing interest in training and deploying generalist CHWs to deliver horizontally integrated packages of essential services, often with an emphasis on prevention, health promotion, and treatment of common illnesses.^34^ Although CHWs are often intended to operate as part of a team (eg, supported by a nurse or medical officer at the local health facility), the persistent low availability of highly trained personnel in rural and low-income areas can potentially lead to an over-reliance on CHWs as de facto primary care providers. In many settings, CHWs are further constrained by employment conditions, inadequate resourcing, limited training, and weak supervision. Generalist CHWs can often be highly cost-effective, but they cannot offer comprehensive services and coordinate care or manage complex multimorbidity. Generalist CHWs are also limited in terms of their ability to tailor clinical care to people’s unique needs and preferences. Some of these limitations also apply to staff tasked with providing first-contact care at local health posts. Despite the heavy investment in CHW-centric models of primary care, with CHW cadres having contributed to remarkable progress for specific health outcomes in some countries, primary care lite—in which the model of care does not offer comprehensiveness, continuity, or coordination—cannot effectively meet the growing burden of multimorbidity or future health system challenges.

At the other end of the spectrum, many countries have strengthened their health systems by integrating primary care specialists (ie, family physicians—doctors with postgraduate family medicine training) to deliver accessible, comprehensive, coordinated, continuous, and person-centred services. These models use family physicians’ specialist training and skills to deliver services that can manage comorbidity, polypharmacy, and less-common conditions, with management tailored according to each person’s unique goals and health needs using a bio-psycho-social approach. Specialist family medicine training often emphasises community engagement, prevention, population health, and curative health services.

Family physicians can work independently (as seen in Iran, Switzerland, Mexico, Greece, and Türkiye) or as part of multidisciplinary teams (as seen in the Netherlands, Costa Rica, Portugal, and the UK). Done right, these primary care teams can offer a panoply of skills with strong integration. However, in the absence of robust processes to ensure continuity, taskification can potentially lead to fragmentation and erosion of any one clinician’s personal responsibility for managing coordination. This set-up is particularly evident in high-income settings.^35^ Although models based on family physicians are associated with high-quality, person-centred, equitable, and cost-effective care,^36^ training enough family physicians to serve every community takes time and considerable investment. Even though technology can magnify the reach and impact of individual practitioners, many countries question whether models based around universal access to a family physician are financially sustainable, given that population demand is rapidly outstripping supply. Moreover, many HICs now face a crisis in primary care not only because of increased demand but also because of an ageing and insufficient primary care physician workforce. The insufficient workforce is a result of medical graduates shunning primary care to opt for other specialities due to low pay and prestige and workload concerns.

Increasing cases of mental ill health, non-communicable diseases, polypharmacy, and multimorbidity mean that we need comprehensive, integrated, bio-psycho-social service delivery with continuity and coordination now more than ever before. In addition to family medicine training for all cadres and the use of multidisciplinary teams and aligned incentives, careful consideration is required to ensure that communities benefit from the traditional skills of family physicians. These skills include the management of complex multimorbidity, holistic management of undifferentiated symptoms, the ability to balance risk and manage the needs of individual patients and the population, and deliver data-driven continuous quality improvement, strengthened by values of community orientation, primary prevention, and engagement with the broad social determinants of health. Looking further ahead, there is an argument for upskilling non-doctor cadres and fully utilising AI tools so that strong primary care (figure) can be made available to all without the need for such high ratios of family physicians, who instead can contribute to care as part of teams serving large populations. We need to be mindful of the fact that growing demand and technological innovation are together disrupting all domains of medicine, including family medicine. In this evolving landscape, we cannot afford to stand still or hew to anachronistic models of service delivery.

**Figure:**
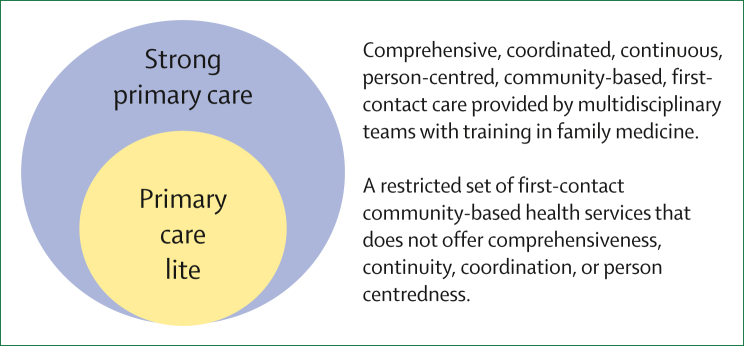
Strong and lite forms of primary care

## Convergent trajectories

High-income, low-income, and middle-income countries appear to be converging towards a similar model of family physician-led multidisciplinary teams in which first contact is made by a CHW, nurse practitioner, or physician assistant. It looks like both HICs and LMICs would like to have family physicians in every community, ideally responsible for a list of patients, providing care alongside their colleagues. In multidisciplinary teams, the family physician would often see patients with more complicated conditions and act as a consultant to other team members.

In many LMICs, this model of family physician-led multidisciplinary teams requires a considerable increase in urban and rural training opportunities for family physicians and the creation of posts across the whole health system, including rural and remote areas. In countries such as Kenya and Botswana, peripatetic family physicians might be responsible for a network of clinics and perform regular visits from their base at the local primary hospital, and over time, the number of physicians will increase. For example, South Africa aims to have a family physician in every subdistrict, community health centre, and primary hospital and can achieve this goal with just 500 additional family physicians.^37^

In HICs, the starting point has been that there is a family physician in every clinic in every community, providing comprehensive services to people presenting with undifferentiated symptoms. As the demand for family physicians is outstripping supply, an increasing number of tasks and responsibilities have started to shift to nurses, allied health professionals, and other health providers. In parts of the UK, family physicians have already stopped providing routine antenatal, ophthalmology, musculoskeletal, mental health, and minor illness care because these roles have been shifted to other cadres or providers, and this fragmentation can disrupt the holistic management and continuity of care.

In both settings, primary care lite might become the focus of our efforts to strengthen and expand primary care as the bulk of first-contact care is shifted towards clinicians with little or no training and capacity to provide comprehensive, continuous, coordinated, person-centred services. Investment in these models are probably essential to achieve UHC when measured in terms of the basic service coverage indicators (eg, getting patients with hypertension onto an antihypertensive medication); however, we would sell ourselves short if community-based delivery of the most basic health services comes to represent our boldest vision for post-2030 primary care. We need to ensure that these contacts wrap essential service delivery with continuity, coordination, and person centredness.

## Future-proofing primary care

As both doctor-centred and CHW-centric models move towards a common middle ground, we should aim to capitalise on the best elements of both models by retaining community orientation and coverage provided by CHWs who are active beyond the clinic doors while leveraging the unique skills of family physicians in the context of multidisciplinary teams to provide high-quality primary care. Primary care providers in Cuba, Thailand, Costa Rica, South Africa, Brazil, Ghent in Belgium, and Westminster and Oxford in the UK already lean heavily into community outreach that can magnify impact (panel 2).^38^, ^39^, ^40^, ^41^, ^42^, ^43^ Although countries such as Thailand and Cambodia show that high-performing primary care teams do not always need to include family physicians, the additional benefit in terms of comprehensiveness indicates that many LMICs still aspire to connect every multidisciplinary team with a family doctor, even if they are remote.^37^^,^^44^, ^45^, ^46^

Panel 2 **Blending community outreach with clinic-based family physician-led care**

According to Brazil’s Family Health Strategy, community health workers (CHWs) are included in neighbourhood-based teams led by a family physician and a nurse. Each team is responsible for a defined population—often around 3000 people—and conducts regular home visits to deliver preventive care, follow-up chronic disease management, and offer social support. This proximity enables high coverage, strong community trust, and early identification of health risks, all underpinned by clinical oversight and continuity of care. Similarly, in South Africa, family physicians based at district hospitals or community health centres often supervise networks of CHWs who deliver care in remote or underserved areas. In the Western Cape, for instance, family physicians can play a strategic role in supporting CHWs through training, data review, and integrated care planning. This approach allows for decentralised access while maintaining clinical governance, facilitating a functional referral system, and ensuring that complex cases are escalated appropriately. In parts of Belgium and the UK, family physicians work alongside CHWs who provide outreach services and help to tackle local social determinants of disease.

AI tools will soon outperform clinicians with respect to medical knowledge, ability to tailor guidelines and evidence to individual patients, diagnostic ability, and possibly even empathy.^47^ Rather than resisting technological advancement, we should focus our efforts on adapting models of care to make best use of these technologies in augmenting the unique skills of each cadre, in the context of a safe and equitable regulatory environment. We need to find the right balance between task sharing and continuity for team-based care and reimagine the role of a family physician.

## Conclusion

It is imperative that we look beyond today's permacrisis to prepare for the challenges of the 2030s and beyond. The principles of Alma-Ata and PHC remain relevant and will continue to be so. Primary care is a crucial platform to deliver equitable access to comprehensive health services that meet the needs of local populations, close to where people live and work, in line with WHO’s foundational principle of health for all. Taking primary care seriously will involve financial and political trade-offs to ensure that health system structures and resources are reoriented around the provision of high-quality family medicine. All societies will need to invest more in their health sectors, and many societies will also need to rebalance spending within the health sector, in line with the recommendations of the WHO Commission on making fair choices on the path to UHC.^48^ The current donor emphasis on primary care lite might get us closer towards universal access to a basket of basic interventions; however, this model is not fit for the future. Meanwhile, both HICs and LMICs are moving towards team-based models of care, led by a (remote) family physician and with community orientation enabled by CHWs. We should ensure that this emerging approach preserves and strengthens the core functions of primary care. The specific cadres involved matter much less than ensuring that the core functions of strong primary care are being delivered in every community. Panel 3 sets out five recommendations to strengthen primary care for the future. Adapting to rapidly transforming societies will require models of care that make the best use of human teams and AI to provide continuity, comprehensiveness, coordination, and person centredness.

**Search strategy and selection criteria**

We reviewed English-language literature and policy documents that were identified through targeted searches of PubMed and grey literature sources, focusing on primary care models, health system reforms, and global health policy frameworks. The searches were conducted on multiple days over 2 months, April and May, 2025, as part of wider internet searches for an evolving set of keywords and terms as different coauthors brought new concepts and ideas to the project. The searches were updated in June, 2025.

Panel 3 **Key priorities for future models of primary care**

• **Reject primary care lite models as the global default**

Delivering a minimalist package of services establishes inequality and fails to meet current and future challenges. Primary care teams should be structured and resourced to support continuity, coordination, comprehensiveness, and person centredness.

• **Family medicine should be foundational and not optional**

Although we acknowledge the current workforce and fiscal constraints, trained family medicine specialists are essential to deliver strong primary care services to meet the challenges of today and tomorrow. Their skillset cannot be easily substituted by community health workers, mid-level staff, or algorithms.

• **Leverage technology but do not replace people**

Artificial intelligence and digital tools can strengthen primary care by augmenting clinicians and enabling data-driven care. However, substituting human clinicians entirely risks deepening disparities, especially for marginalised populations.

• **Hybrid models probably offer the best path forward**

Health systems, especially those focused on addressing the social determinants of health, should aim to extend community outreach roles while embedding workers within team-based structures that maintain quality and coordination with input from clinicians trained in family medicine.

• **Systemic investment and reforms are needed**

Structural investment in governance, financing, facilities, supplies, and digital infrastructure is as important as workforce expansion for future-ready primary care.

## Declaration of interests

We declare no competing interests.
